# Establishment of a Latin American dataset to enable the construction of gestational weight gain charts for adolescents

**DOI:** 10.1371/journal.pone.0296981

**Published:** 2024-01-26

**Authors:** María Victoria Benjumea Rincón, Sandra Lucía Restrepo-Mesa, Thais Rangel Bousquet Carrilho, Gilberto Kac, Eduardo Atalah Samur, Josué Santiago Cano Pulgarín, Alejandro Estrada Restrepo, Cristian David Santa Escobar

**Affiliations:** 1 University of Antioquia, Medellín, Colombia; 2 Research Group on Food and Human Nutrition, School of Nutrition and Dietetics, University of Antioquia, Medellin, Colombia; 3 Josué de Castro Nutrition Institute, Nutritional Epidemiology Observatory, Federal University of Rio de Janeiro, Rio de Janeiro, Brazil; 4 Faculty of Medicine, University of Chile, Santiago, Chile; 5 Research Group on Demographics and Health, School of Nutrition and Dietetics, University of Antioquia, Medellín, Colombia; Universidade Federal do Maranhão: Universidade Federal do Maranhao, BRAZIL

## Abstract

Gestational weight gain is an important indicator for monitoring nutritional status during pregnancy. However, there are no gestational weight gain references created for adolescents or national datasets to enable the construction of such graphs up to date. This manuscript aims to describe the creation of a Latin American dataset to construct gestational weight gain references for adolescents aged 10–19 years old. Gestational weight gain data from studies conducted in nine countries (Argentina, Brazil, Chile, Colombia, Mexico, Panama, Paraguay, Peru, and Uruguay) collected between 2003 and 2021 were harmonized. Data on height, weight, and gestational age in at least two gestational trimesters were included. Pregnant adolescents should be free of diseases that could affect weight, and newborns should weigh between 2,500–4,000 g and be free of congenital malformations. The final dataset included 6,414 individuals after data cleaning. Heterogeneity between the countries was assessed by calculating standardized site differences for GWG and *z* scores of height-for-age. Several imputation procedures were tested, and approximately 10% of the first-trimester weights were imputed. The prevalence of individuals with underweight (1.5%) and obesity (5.3%) was low, which may lead to problems when modeling the curves for such BMI categories. Maternal height and gestational weight gain did not show significant differences by country, according to the standardized site differences. A harmonized dataset of nine countries with imputed data in the first trimester of pregnancy was prepared to construct Latin American gestational weight gain curves for adolescents.

## Introduction

Anthropometry is a suitable technique to assess adolescent individuals’ growth and nutritional status [1]. To date, there are no guidelines for monitoring gestational weight gain (GWG) during the prenatal care of adolescent mothers [2,3]. In Latin America, GWG references based on data from adult pregnant individuals from Chile, Argentina, Uruguay, or the United States have been used interchangeably on adolescents [4,5]. However, such references designed for adults are not recommended for adolescent use due to differences in this age group’s physiological condition and social determinants of health and nutrition [4,6,7].

Experts have previously recommended that anthropometric references designed to assess GWG should result from longitudinal studies of selected populations with a low incidence of maternal and fetal complications [8]. Additionally, these references should consider the pre-pregnancy body mass index (BMI) status [8,9]. In those studies, anthropometric measurements should be collected before and during pregnancy and childbirth [10]. There is no comprehensive dataset to construct such charts for adolescents in Latin American countries. To the best of our knowledge, there are no initiatives in those countries to explore GWG data of adolescents and particular characteristics these groups may have, such as late prenatal care initiation and continuing height growth during pregnancy.

Thus, this study aims to describe the efforts for data acquisition, harmonization, and several methodological challenges faced when creating a dataset to enable the construction of GWG charts for adolescents living in Latin America.

## Materials and methods

### Study design

This is a retrospective longitudinal study based on the secondary analysis of harmonized datasets from nine Latin-American countries (Argentina, Brazil, Chile, Colombia, Mexico, Panama, Paraguay, Peru, and Uruguay). Researchers and officials from health ministries or institutions were invited to participate in the initiative and provide datasets for analyses. Participants were selected based on their previous publications in the field and institutional affiliations.

A standardized form was used to gather the necessary data from each study and decide their potential inclusion in the combined dataset. Each invited researcher provided the following details about their studies: the origin of the study, city and country of data collection, source of data collection (primary or secondary), the sample size of the dataset, maternal age/date of birth, the total number of prenatal care visits for each individual, presence of obstetric and previous diseases (such as diabetes mellitus, hypertension), weight and height measurements, gestational age at prenatal care visits, and birth weight.

The forms were evaluated by the core team of researchers of the project, and the datasets from studies that included the variables necessary for the construction of the charts were requested. Over 30 people from 13 countries were invited, and those from the nine countries listed above answered the form and were included in this project. Then, the data of 33,446 pregnant individuals with approximately 150,000 weight measurements were consolidated, and the eligibility criteria were applied. These 150,000 measurements referred to individuals who gave birth to a child alive without congenital malformations.

### Eligibility criteria

To be included in the study, adolescents had to: be between 10 and 19 years old (verified by date of birth and conception); have at least one prenatal weight (kg) in the second and third trimesters, with their respective gestational age (weeks); give birth at term (37 to 42 weeks), and with birth weight between 2,500 and 4,000 g. Individuals also needed to be free of hypertension, preeclampsia, diabetes mellitus or gestational diabetes, tuberculosis, or cardiovascular diseases. Adolescents with height-for-age <-2 *z* score of the WHO charts were classified as having stunting [1] and were removed from the analyses. It is worth saying that only pregnant individuals without missing data in the variables of interest (maternal age, pre-pregnancy weight, height, pre-pregnancy BMI, cumulative GWG in each visit, gestational age in each, height-for-age, birth weight and length) were included.

All selected datasets were individually and carefully revised during the data-cleaning process. The pregnant adolescents with GWG ≥ 30 kg and those with an impossible biological weight gain trajectory were excluded. This was manually identified by checking the sequence of the adolescent’s weights during pregnancy. The weight gain of each adolescent was graphed, the weights with errors were identified, and weight gain or losses that did not follow the pattern observed for that particular pregnant individual were removed (S1 Fig). Finally, we removed individuals who did not have pre-pregnancy weight or BMI data. At the end of this process, data from 6,414 adolescent pregnant individuals and 34,943 weight records were available to construct the GWG charts.

### Main variables

Maternal age (years) was estimated as the difference between the date of birth of the pregnant individual and the date of conception. The date of the conception was determined based on an algorithm from the ‘ob Wheel’ available at https://obwheel.quartertone.net/.

Pre-pregnancy weight was obtained from three different sources, i.e., measured, abstracted from medical records, or self-reported. Height was measured at enrollment in the study or the start of prenatal care. These two variables were used to calculate pre-pregnancy BMI (kg/m^2^). Pre-pregnancy BMI was classified according to the WHO charts z scores as underweight, <-2 Standard Deviation (SD); normal weight, ≥ -2 SD to ≤ +1 SD; overweight, > +1 SD to ≤ +2SD; and obesity, > +2 SD [1]. Cumulative GWG (kg) in each visit was calculated as the difference between the weight in each visit and the pre-pregnancy weight.

Gestational age in each visit was already available in the datasets and was not recalculated. Height-for-age *z* scores were calculated using the WHO AnthroPlus software. Birth weight (g) and length (cm) were also available in each dataset, and it was not possible to know the origin of those measurements (i.e., if they were measured in the study, abstracted from medical records, or reported by the mother).

### Statistical analysis

#### Heterogeneity assessment

The heterogeneity of GWG and height-for-age *z* scores according to the pre-pregnancy BMI category across the nine countries was assessed by calculating the Standardized Site Differences (SSD). This method consists of the calculation of *z* scores for the means of GWG in gestational age groups (0–7, 8–14, 15–21; 22–28; 29–35; 36–42 weeks) and *z* scores of height-for-age in relation to the pooled mean and SD in each group/age [11]. According to Cohen [12], differences of 0.2 SD units are considered small, 0.5 SD units are acceptable, and 0.8 are large. Thus, SSD values within the range of ± 0.5 units were considered homogeneous, representing that the data could be combined in a unique dataset to construct the charts.

We also performed a sensitivity analysis for the heterogeneity assessment of GWG, excluding studies with less than ten records from the selected age groupings. This procedure was necessary to evaluate if smaller datasets could contribute highly to the observed heterogeneity due to the sample size and not to true differences between the GWG of the adolescents from those countries.

#### First-trimester missing data

The distribution of GWG was evaluated according to gestational age for each pre-pregnancy BMI category. Approximately 10% of GWG records did not present values between the fifth and thirteen weeks of pregnancy (first trimester). This proportion of missing data may lead to problems when modeling the GWG curves. For this reason, we decided to impute the weight in this period. We assumed that the data was missing not at random (MNAR). This decision considered that the lack of weight measurement in this period was related to a late beginning of prenatal care, which could not be explained by other variables available in the dataset. Several statistical techniques to deal with this issue were tested.

First, an initial data point for individuals with missing data was defined for a random week between 5 and 13. Subsequently, imputation methods were applied for univariate missing data (weight in the randomly selected week). These methods were chosen considering their adaptations to deal with MNAR. Predictive mean matching [13], random forest [14], classification and regression trees [15], random indicator [16], and imputation under the normal linear model with bootstrap [17] were considered. This later procedure was selected. The method calculates univariate imputations by drawing a bootstrap sample from the complete part of the data (individuals with first-trimester weight values) and subsequently taking the least-squares estimates from the linear model given the bootstrap sample, which incorporates the variability of the sampling into the parameters. It does not include the other weight measurements the individual may have, but it is possible to include other variables in the regression model [17]. The adolescent’s id, gestational age at each antenatal visit (weeks), and the number of antenatal visits during pregnancy were included as covariates in the normal linear model. We performed 50 iterations with five imputation datasets (m = 5), and Rubin’s rules were used to combine the estimates [18]. We constructed graphs using generalized additive models of location, shape and scale (GAMLSS) [19] for each imputation method to compare the distribution of the first-trimester GWG according to gestational age. Two criteria were used to select the best imputation method. The first was the statistical criterion, in which the fit of the models was evaluated with the GAIC, AIC and Global Deviance [20] indicators, additionally the assumptions of normality and homoscedasticity were verified, and for the specification, the quantile comparison was implemented [21]. The second criterion was a panel of experts in prenatal nutrition that assessed the biological plausibility of the data obtained in each estimated model, and analyzed each of the data of the pregnant woman in the three trimesters of pregnancy; in this case, special emphasis was placed on the behavior of the negative weight gains between trimesters and on the extent of this weight loss during the course of the pregnancy (between I and III trimester), and the smoothing of the curves in the first trimester and the amplitude of the percentile channels were also considered.

#### Descriptive analyses

Medians and interquartile ranges for continuous variables were calculated according to pre-pregnancy BMI category, country, antenatal visits, and trimesters. The analyses were performed in Python v3.8.2 and R v4.0.3.

#### Ethics

This study was approved by the Bioethics Committee of the Faculty of Dentistry of the Universidad de Antioquia, act number 12 of October 18, 2019. Only de-identified data was used in the analyses, which were performed by authorized investigators. The principal investigator of the University of Antioquia signed a confidentiality and custody agreement for the data with the investigator or institutional representative of each country.

## Results

The data included in this analysis were collected between 2003 and 2021 and came from different sources: 13.7% from research projects, 68.3% from information systems, and 18.0% from maternal and perinatal care institutions’ medical records (S1 Table in S1 File).

**Fig 1:**
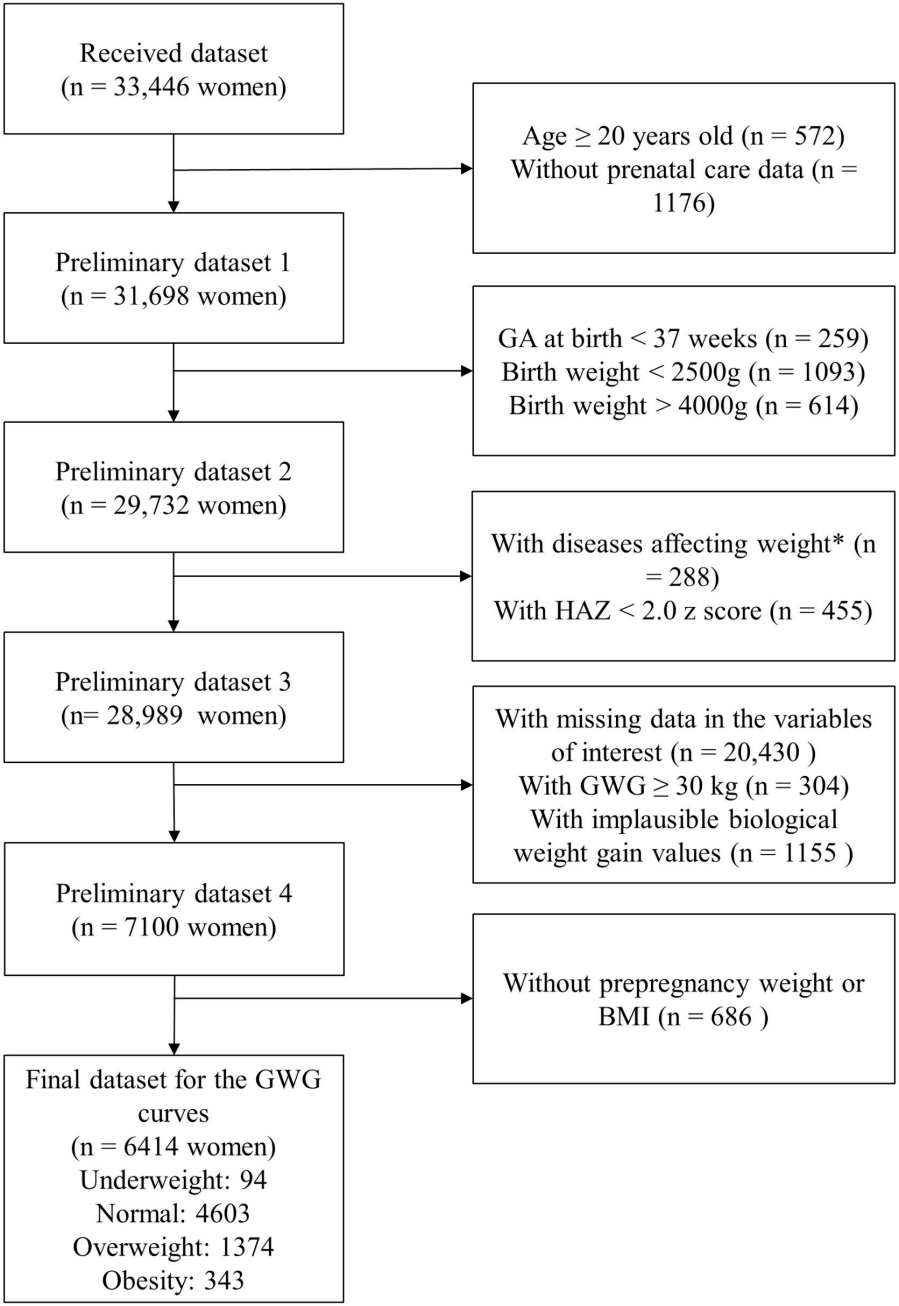
Flowchart for the constitution of the final dataset. Notes: Diseases considered: Chronic hypertension or hypertensive disorders during pregnancy, diabetes mellitus or gestational diabetes, tuberculosis, or cardiovascular diseases. Abbreviations: BMI: Body mass index; GA: Gestational age; GWG: Gestational weight gain; HAZ: Height-for-age z score.

The harmonized dataset (n = 6,414 pregnant adolescents) comprised mainly adolescents from Uruguay (36.6%), and Panamá (31.8%) (S1 Table in S1 File). Most adolescents were between 14–17 years old (50.9%). The prevalence of individuals classified with pre-pregnancy underweight was 1.5% and obesity 5.3%, while normal weight and overweight were 71.8% and 21.4%, respectively (Fig 1).

Height, pre-pregnancy weight, and third-trimester weight varied between the countries. Adolescents from Brazil, Chile, and Uruguay showed the highest median heights compared to those from Panamá and Peru. The median pre-pregnancy and third-trimester weights were higher for Chilean individuals and smaller for Peruvians compared to the other countries. The median birth weight was lower among Mexican adolescents (Table 1).

**Table 1:** Distribution of the variables of interest for adolescents from nine Latin American countries (n = 6,414 individuals).

Country	n	Maternal age (years)	Pre-pregnancy BMI (kg/m^2^)	Height (cm)	Pre-pregnancy weight (kg)	Third-trimester weight (kg)	Birth weight (g)	Gestational age at birth (weeks)
		Median (IQR)						
Argentina	545	15 (14–16)	21.6 (19.7–23.6)	155 (152–159)	53.3 (48.2–59)	65 (60–71.8)	3,220 (3000–3450)	39 (38–40)
Brazil	254	17 (16–18)	20.9 (19.4–23.0)	159 (156–163)	55.5 (50.7–61)	67.5 (62.–74.8)	3,190 (2950–3472)	39 (38–40)
Chile	324	18 (17–19)	23.2 (21.1–25.7)	158 (155–162)	60 (54–68)	72 (65–79)	3,275 (3094–3526)	39 (38–40)
Colombia	295	18 (17–19)	21.8 (20.0–24.1)	157 (154–162)	55.5 (50–62)	65 (59.5–72.6)	3,180 (2980–3385)	39 (38–40)
México	119	16 (15–17)	21.8 (20.5–23.6)	155 (151–158)	56.4 (51.3–63)	64.5 (59.6–70.4)	3,045 (2799–3240)	39 (38–40)
Panama	2,042	18 (16–19)	23.4 (21.2–26.0)	153 (150–158)	56 (51–62)	63.8 (58.5–70.2)	3,173 (2930–3425)	39 (38–40)
Paraguay	396	18 (17–18)	22.0 (19.9–24.5)	157 (154–160)	56.5 (51–63.5)	65 (58.88–72)	3,250 (3000–3500)	39 (38–40)
Peru	91	16 (15–17)	21.9 (19.8–23.6)	153 (150–156)	54 (50–59.5)	62 (57.0–68.5)	3,280 (3070–3520)	39 (39–40)
Uruguay	2,348	18 (17–18)	21.1 (19.3–23.1)	159 (155–162)	55.7 (50.8–61.5)	66.1 (60.38–73)	3,260 (3000–3505)	39 (38–40)

Note: BMI: Body mass index.

**Fig 2:**
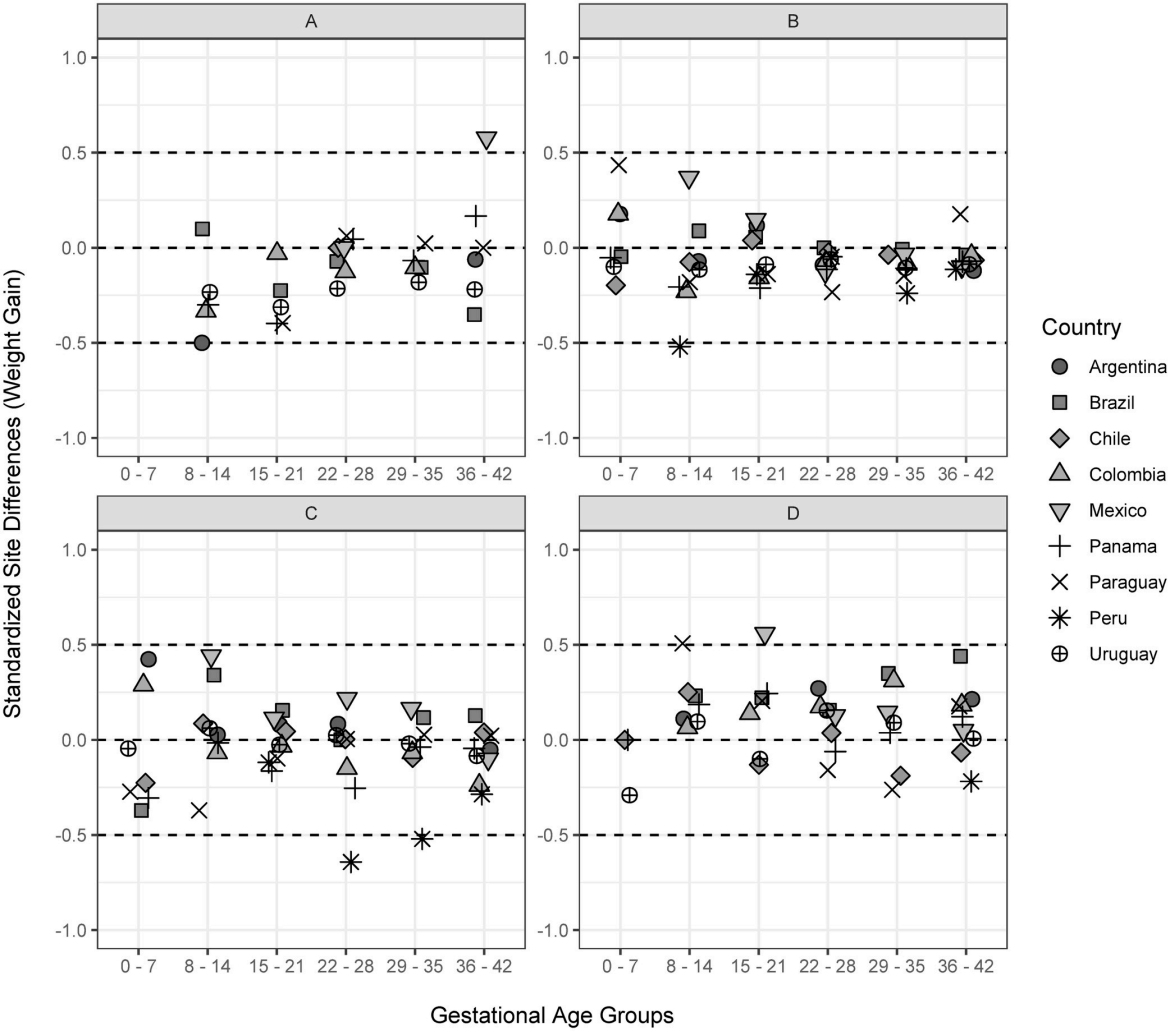
Standardized Site Differences (SSD) for gestational weight gain (kg) according to country and gestational age group (weeks). A. underweight (BMI/age <-2 SD); B. normal-weight (BMI/age ≥ -2 SD and ≤ +1 SD); C. overweight (BMI/age > +1 SD and ≤ +2SD); D. obesity (BMI/age > +2 SD).

**Fig 3:**
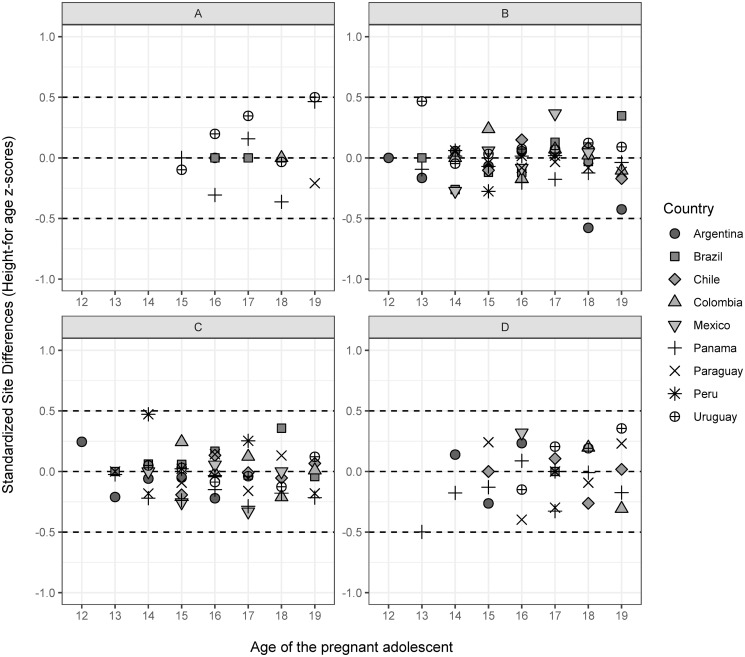
Standardized Site Differences and size (SSD) for height-for-age *z* scores according to country and age (years). A. underweight (BMI/age <-2 SD); B. normal-weight (BMI/age ≥ -2 SD and ≤ +1 SD); C. overweight (BMI/age > +1 SD and ≤ +2SD); D. obesity (BMI/age > +2 SD).

No heterogeneity was observed for GWG according to gestational age and height-for-age z scores (Figs 2 and 3). Most of the SSD values for both indicators fell between -0.5/+0.5. For GWG, the SSDs were farther from the -0.5/+0.5 interval for individuals with underweight from Mexico at 36–42 weeks; for those with overweight from Peru at 22–28 weeks, and those with obesity from Mexico at 15–21 gestational weeks. When removing the countries with < 10 observations in each selected gestational age interval, it was possible to see that all the values fell in the expected range, suggesting that the low sample size could contribute to the observed heterogeneity (S2 Fig). Based on that, we decided not to remove the observations of the countries with low sample sizes.

For the first trimester, individuals from Colombia classified with normal-weight, overweight, and obesity had the lower median (0 kg) GWG than the other countries. A negative median GWG was observed for individuals with underweight from Argentina (-2.0 kg). Several countries did not have any GWG data in this period for some BMI categories (S2 Table in S1 File).

Chile, Peru, and Mexico had a very low sample size available for several BMI categories for the second trimester. For individuals with underweight, the lowest median GWG was observed in Chile (2.8 kg). For normal weight, the median GWG for Panamá (0.7 kg) was the lowest, and for overweight and obesity, the lowest median (0 kg) was observed for adolescents from Peru and Panamá, respectively (S2 Table in S1 File).

In the third pregnancy trimester, the countries with the median GWG lowest for BMI category were: Panama in normal weight (8.4 kg), Panama and Paraguay in overweight (both 7kg), and Paraguay in obesity (6kg). Adolescents with underweight from Brazil and Paraguay presented the highest median GWG (17.2 and 17.0 kg, respectively) (S2 Table in S1 File).

**Fig 4:**
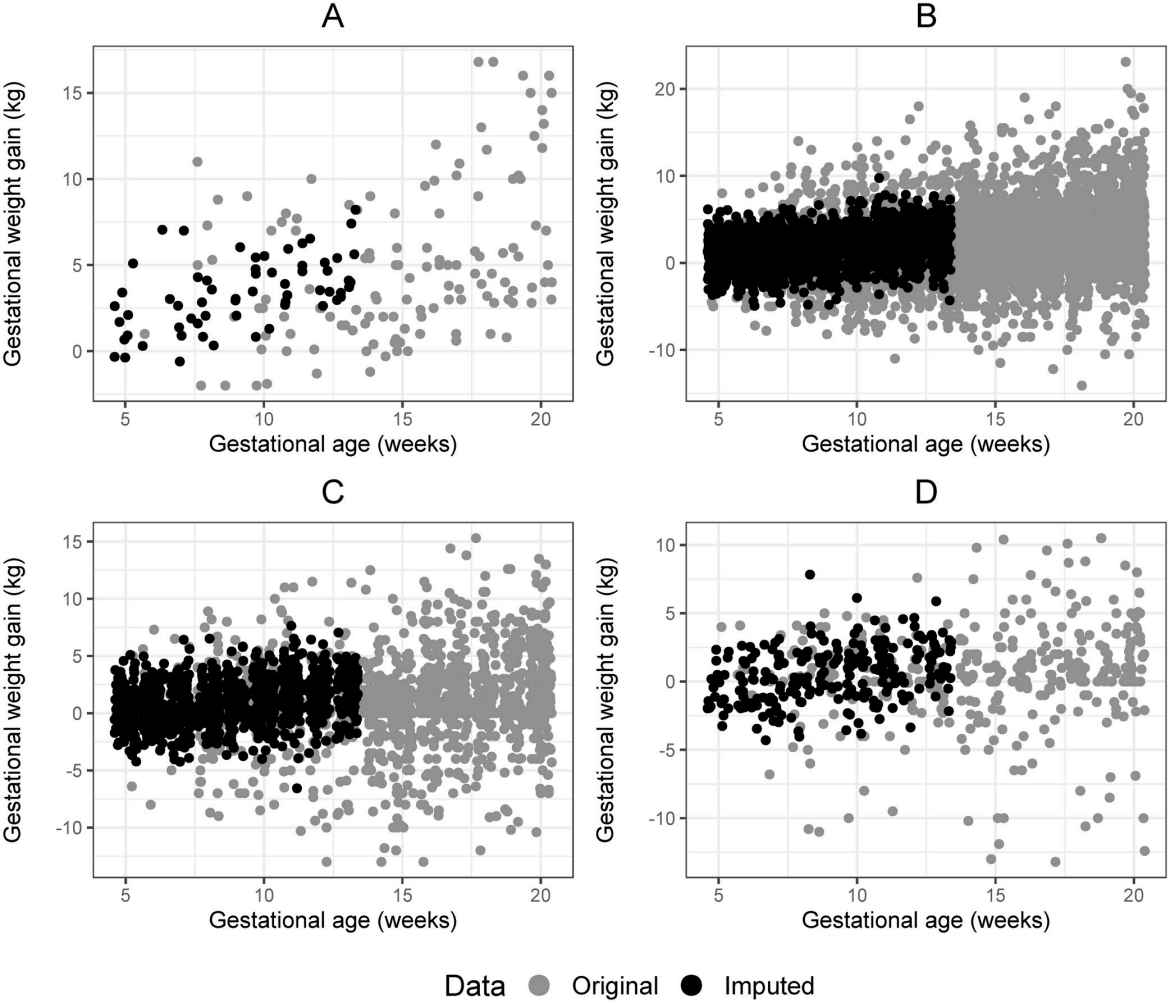
Distribution of gestational weight gain according to gestational age in early pregnancy for each pre-pregnancy BMI before and after the imputation procedure. A. underweight (BMI/age <-2 SD); B. normal weight (BMI/age ≥ -2 SD and ≤ +1 SD); C. overweight (BMI/age > +1 SD and ≤ +2SD); D. obesity (BMI/age > +2 SD).

The imputation process was made only for the underweight and overweight categories because, in the rest of the categories, the models presented adequate adjustments. The proportion of missing data in the first trimester was similar across the pre-pregnancy BMI categories and varied from 10.4% (underweight) to 12.8% (overweight). The imputation procedure completed the distribution of weight gain in the first trimester (between 5–13 weeks) with values within the expected range for the period (Fig 4). When the median of GWG in the first trimester was compared to the values after imputation, it was possible to observe that the values imputed were very similar to those originally observed in each country (S3 Table in S1 File).

## Discussion

The final dataset compiled in this project from nine Latin American countries results from a rigorous data harmonization process. The homogeneity of the GWG and height-for-age *z* scores and similar maternal age distributions across the included countries reinforce the possibility of combining the data into a single dataset to construct GWG graphs for adolescents aged 10–19 years.

This study revealed that approximately 10.2% of the pre-pregnancy weight was missing. The high proportion of missing GWG data in the first trimester of pregnancy represented a challenge when constructing the GWG charts and needed to be adequately treated. We tested several methods adapted to deal with MNAR and decided to use imputation under the normal linear model with bootstrap to create first-trimester measurements used to calculate GWG in this period. The lack of data in the first trimester is recurrent. Yang et al. [22] also proposed an imputation approach to deal with missing first-trimester weight data but considered only imputing weight in a specific week (the 9^th^ gestational week). Imputing a specific week would not solve the need for data throughout the first trimester to construct the charts. Other authors designing GWG curves for adults also faced this methodological problem and decided not to impute a first-trimester weight measurement [23,24].

The absence of data for GWG in the first trimester is consistent with previous studies from Latin America that show worrying figures on the late attendance of adolescents to the first prenatal care visit [25–27], the low coverage of prenatal care programs in the region [28], and the possible difficulties for timely access to health services [29,30]. Each government must establish goals for monitoring the quality and quantity of the data collected in each trimester of pregnancy, and special attention must be paid to adolescents.

Creating GWG curves requires enough sample size distributed according to all pre-pregnancy BMIs categories. Therefore, the low prevalence of adolescents classified as underweight (1.5%) and obesity (5.3%) according to pre-pregnancy BMI is worth mentioning. These prevalences are lower than those observed by Samano et al. [31] in Mexico (5.0 and 10.1%, respectively, using the WHO criteria). Pregnant adolescents with underweight and obesity, especially adolescents, are usually considered at a higher risk for adverse outcomes and are monitored in specialized prenatal care services. Therefore, they might not have been captured in the original studies that comprised the dataset used in the current investigation. Additionally, because the aim is to construct prescriptive GWG curves, individuals who gave birth to neonates with weight outside the 2,500–4,000g interval had to be removed. Adopting these criteria may have reduced the available sample size in these two BMI categories, but including them would result in an inappropriate dataset for constructing such curves.

The prevalence of adolescents in each BMI category in the harmonized dataset is similar to values reported for adults in Latin America, especially regarding normal and overweight. In Colombia, Benjumea and Bermúdez [5] reported a prevalence of 45.8% for normal and 24.7% for overweight. In Mexico, when comparing the pre-pregnancy BMI of adolescent mothers using three different classifications, it was observed that 73.4% of the individuals were classified with normal weight, which is very close to the prevalence observed in the current study (71.8%) [31].

The values for GWG in the first (before imputation) and second trimesters in most countries included in this study are lower than those observed for adults in other Latin American countries. Among adult pregnant individuals registered in a Brazilian surveillance system, Carrilho et al. [32] observed that, in 2018, the mean GWG varied between 0.8–1.7 kg in the first trimester and 3.8–6.0 kg in the second trimester. Several countries had a median GWG = 0 kg in the current study until the second pregnancy trimester. For the third-trimester GWG, the values observed in the harmonized dataset are similar to those reported for total GWG for adults by Wang et al. [33]. The authors used data from the Demographic Health Surveys to estimate the total GWG for 2015 for several regions. The projected mean for Latin American and Caribbean countries was 11.8 kg (95% uncertainty range 6.2–17.4 kg). This interval includes all the medians observed in the current study for GWG in the third trimester for all BMI categories. The low median GWG observed in the first and second trimesters and the values for the third trimester closer to the total recommended GWG for adults suggest that these adolescents have a different weight gain pattern during pregnancy, with higher rates in the third and not the second pregnancy trimester, as it is usually expected for adults pregnant [9].

### Strengths and limitations

This is the first study in Latin America to combine datasets from several countries to create a robust pooled dataset to analyze GWG among adolescents and the first initiative to construct GWG charts for this group. Several GWG curves for adults have been created since 2013 [23,24,34,35], and individuals < 19 years old were excluded from all of them. The rigorous harmonization of the dataset, with conferences by the team of researchers, continuous discussions, and visual inspections of the data are strengths of this study.

However, some limitations need to be pointed out. One of the main challenges in constructing this dataset was the non-existence of databases in some Latin-American countries and the low quality of data from national information systems, highlighting the need to implement and standardize adequate perinatal systems. Thus, it is difficult to evaluate the generalizability of the final dataset because national representative studies with adolescents to compare their characteristics are scarce. However, it is important to mention that we did not aim to have a representative sample of adolescent individuals from each selected country or the Latin-American continent.

The lack of data in the first trimester, which can reflect a feature of this group, is a significant limitation, which we tried to overcome with multiple imputation techniques. These methods can be extended to similar situations in this field.

Due to the different data sources used to construct the harmonized dataset and the lack of information, it is not possible to know the source pre-pregnancy weight data used (measured, abstracted from medical records, or self-reported) and the period to which the pre-pregnancy weight refers to, i.e., immediately before pregnancy, six months before, a year, etc. Although self-reported pre-pregnancy weight has a high agreement with measured first-trimester weight in Brazil and could be used to calculate pre-pregnancy BMI and GWG [36], the quality of other types of pre-pregnancy weight is unknown. The source of weight and length at birth data used is unknown too. The lack of data on sociodemographic characteristics of adolescents is also a limitation when working with a dataset resulting from a combination of multiple sources.

Finally, the absence of data on the height at the end of pregnancy did not allow us to evaluate how growth continues (or does not) to occur and its impact on GWG. This is a fundamental point when working with pregnancy in adolescence, especially at younger ages. Future studies with this group could incorporate at least one height evaluation at the end of pregnancy to allow for that possibility.

## Conclusion

The main outcome of this study is a harmonized and homogeneous dataset from nine countries, with imputed data in the first pregnancy trimester, prepared for constructing Latin American GWG charts for adolescents. This is the first Latin American initiative to consolidate a dataset of pregnant adolescents that overcame problems in data harmonization, such as identifying implausible values, the assessment of heterogeneity, and the high proportion of missing data in the first pregnancy trimester. In Latin America, the information on the nutritional status of pregnant adolescents is limited, and the quality of the data from national information systems is far from ideal; however, with this rigorous harmonization process, we were able to obtain a large international dataset that could be used to construct unprecedented GWG curves for this group.

## Supporting information

S1 Fig Example two women’s weight gain trajectory with measurements flagged as implausible (outliers). (TIF)

S2 Fig Standardized Site Differences (SSD) for gestational weight gain (kg) according to country and gestational age group (weeks) removing datasets with n ≤ 10 in the selected time intervals: A. underweight (BMI/age <-2 SD); B. normal weight (BMI/age ≥ -2 SD and ≤ +1 SD); C. overweight (BMI/age > +1 SD and ≤ +2SD); D. obesity (BMI/age > +2 SD).
(TIF)

S1 File Supplementary tables. (DOCX)

## References

[1] de Onis M, Onyango AW, Borghi E, Siyam A, Nishida C, Siekmann J. Development of a WHO growth reference for school-aged children and adolescents. Bull World Health Organ. 2007;85(9):660–7. doi: 10.2471/blt.07.043497 18026621 PMC2636412

[2] Groth S. Are the Institute of Medicine recommendations for gestational weight gain appropriate for adolescents? J Obstet Gynecol Neonatal Nurs. 2007;36(1):21–7. doi: 10.1111/j.1552-6909.2006.00117.x 17238943

[3] Pinho-Pompeu M, Paulino DSM, Morais SS, Crubelatti MY, Pinto ES JL, Surita FG. How to classify BMI among pregnant adolescents? A prospective cohort. Public Health Nutr. 2019;22(2):265–72. doi: 10.1017/S1368980018002768 30378516 PMC10277188

[4] Benjumea MV. [Diagnostic accuracy of five gestational references to predict insufficient birth weight]. Biomedica. 2007;27(1):42–55. 17546223

[5] Ministerio de Salud y Protección Social, Departamento Administrativo para la Prosperidad Social, Instituto Colombiano de Bienestar Familiar (ICBF), Instituto Nacional de Salud (INS), Universidad Nacional de Colombia. Encuesta Nacional de la Situación Nutricional (ENSIN) en Colombia 2015. Bogotá. 2019.

[6] Amaral J de F, Vasconcelos GM, Torloni MR, Fisberg M, Sampaio I de P, Guazzelli CA. Nutritional assessment of pregnant adolescents: comparison of two popular classification systems. Matern Child Nutr. 2015;11(3):305–13. doi: 10.1111/mcn.12016 23230989 PMC6860249

[7] Federación Colombiana de Asociaciones de Perinatología (FECOPEN). Embarazo de Alto Riesgo. 2021. https://www.fecopen.org/images/Embarazo_de_Alto_Riesgo.pdf.

[8] Ohadike CO, Cheikh-Ismail L, Ohuma EO, Giuliani F, Bishop D, Kac G, et al. Systematic review of the methodological quality of studies aimed at creating gestational weight gain charts. Adv Nutr. 2016;7(2):313–22. doi: 10.3945/an.115.010413 26980814 PMC4785472

[9] Institute of Medicine (U.S.). Committee to Reexamine IOM Pregnancy Weight Guidelines. Weight gain during pregnancy: reexamining the guidelines. Washington, DC: National Academies Press; 2009. xiv, 854 p. p.

[10] WHO Expert Committee on Physical Status: the Use and Interpretation of Anthropometry. Physical status: the use and interpretation of anthropometry. Geneva: World Health Organization; 1995. x, 452 p. p. 8594834

[11] Borghi E, de Onis M, Garza C, Van den Broeck J, Frongillo EA, Grummer-Strawn L, et al. Construction of the World Health Organization child growth standards: selection of methods for attained growth curves. Stat Med. 2006;25(2):247–65. doi: 10.1002/sim.2227 16143968

[12] Cohen J. Statistical Power Analysis for the Behavioral Sciences: Taylor & Francis; 2013.

[13] Morris TP, White IR, Royston P. Tuning multiple imputation by predictive mean matching and local residual draws. BMC Med Res Methodol. 2014;14:75. doi: 10.1186/1471-2288-14-75 24903709 PMC4051964

[14] Shah AD, Bartlett JW, Carpenter J, Nicholas O, Hemingway H. Comparison of random forest and parametric imputation models for imputing missing data using MICE: a CALIBER study. Am J Epidemiol. 2014;179(6):764–74. doi: 10.1093/aje/kwt312 24589914 PMC3939843

[15] Doove LL, Van Buuren S, Dusseldorp E. Recursive partitioning for missing data imputation in the presence of interaction effects. Computational Statistics & Data Analysis. 2014;72:92–104.

[16] Jolani S, Frank LE, van Buuren S. Dual imputation model for incomplete longitudinal data. Br J Math Stat Psychol. 2014;67(2):197–212. doi: 10.1111/bmsp.12021 23909566

[17] van Buuren S. Flexible Imputation of Missing Data. Second Edition: CRC Press; 2018.

[18] Rubin DB. Multiple Imputation for Nonresponse in Surveys: Wiley; 1987.

[19] Stasinopoulos DM, Rigby RA. Generalized Additive Models for Location Scale and Shape (GAMLSS) in R. Journal of Statistical Software. 2007;23(1): 1–46.

[20] Bozdogan H. Akaike’s Information Criterion and Recent Developments in Information Complexity. Journal of Mathematical Psychology. 2000;44(1): 62–91. doi: 10.1006/jmps.1999.1277 10733858

[21] Rigby R, Stasinopoulos D, Voudouris V. Discussion: A comparison of GAMLSS with quantile regression. Statistical Modelling. 2013;13(4): 335–348.

[22] Yang J, Wang D, Darling AM, Liu E, Perumal N, Fawzi WW, et al. Methodological approaches to imputing early-pregnancy weight based on weight measures collected during pregnancy. BMC Med Res Methodol. 2021;21(1):24. doi: 10.1186/s12874-021-01210-3 33546607 PMC7863454

[23] Ismail LC, Bishop DC, Pang R, Ohuma EO, Kac G, Abrams B, et al. Gestational weight gain standards based on women enrolled in the Fetal Growth Longitudinal Study of the INTERGROWTH-21st Project: a prospective longitudinal cohort study. BMJ. 2016;352:i555. doi: 10.1136/bmj.i555 26926301 PMC4770850

[24] Kac G, Carrilho TRB, Rasmussen KM, Reichenheim ME, Farias DR, Hutcheon JA, et al. Gestational weight gain charts: results from the Brazilian Maternal and Child Nutrition Consortium. Am J Clin Nutr. 2021;113(5):1351–60. doi: 10.1093/ajcn/nqaa402 33740055 PMC8106749

[25] Atienzo EE, Suárez-López L, Meneses-Palomino M, Campero L. Características de la atención prenatal en adolescentes del Perú, comparación con mujeres adultas. Revista Medica Herediana. 2016;27:131–8.

[26] Cunnington AJ. What’s so bad about teenage pregnancy? J Fam Plann Reprod Health Care. 2001;27(1):36–41. doi: 10.1783/147118901101194877 12457546

[27] Debiec KE, Paul KJ, Mitchell CM, Hitti JE. Inadequate prenatal care and risk of preterm delivery among adolescents: a retrospective study over 10 years. Am J Obstet Gynecol. 2010;203(2):122 e1–6. doi: 10.1016/j.ajog.2010.03.001 20471628

[28] Banco Interamericano de Desarrollo (BID). Cobertura y oportunidad de la atención prenatal en mujeres pobres de 6 países de Mesoamérica. 2017. https://publications.iadb.org/es/cobertura-y-oportunidad-de-la-atencion-prenatal-en-mujeres-pobres-de-6-paises-de-mesoamerica.

[29] Mendoza Tascón LA, Arias Guatibonza MD, Peñaranda Ospina CB, Mendoza Tascón LI, Manzano Penagos S, Varela Bahena AM. Influencia de la adolescencia y su entorno en la adherencia al control prenatal e impacto sobre la prematuridad, bajo peso al nacer y mortalidad neonatal. Revista Chilena de Obstetricia y Ginecología. 2015;80:306–15.

[30] Reynolds HW, Wong EL, Tucker H. Adolescents’ use of maternal and child health services in developing countries. International family planning perspectives. 2006;32(1):6–16. doi: 10.1363/3200606 16723297

[31] Samano R, Chico-Barba G, Martinez-Rojano H, Godinez E, Rodriguez-Ventura AL, Avila-Koury G, et al. Pre-pregnancy body mass index classification and gestational weight gain on neonatal outcomes in adolescent mothers: A follow-up study. PLoS One. 2018;13(7):e0200361. doi: 10.1371/journal.pone.0200361 30001386 PMC6053897

[32] Carrilho TRB, Rasmussen KM, Hutcheon JA, Alves RFS, Farias DR, Freitas-Costa NC, et al. Prevalence and temporal trends in prepregnancy nutritional status and gestational weight gain of adult women followed in the Brazilian Food and Nutrition Surveillance System from 2008 to 2018. Matern Child Nutr. 2021:e13240. doi: 10.1111/mcn.13240 34258876 PMC8710119

[33] Wang D, Wang M, Darling AM, Perumal N, Liu E, Danaei G, et al. Gestational weight gain in low-income and middle-income countries: a modelling analysis using nationally representative data. BMJ Glob Health. 2020;5(11). doi: 10.1136/bmjgh-2020-003423 33177038 PMC7661366

[34] Hutcheon JA, Platt RW, Abrams B, Himes KP, Simhan HN, Bodnar LM. Pregnancy weight gain charts for obese and overweight women. Obesity. 2015;23(3):532–5. doi: 10.1002/oby.21011 25707378 PMC4340088

[35] Hutcheon JA, Platt RW, Abrams B, Himes KP, Simhan HN, Bodnar LM. A weight-gain-for-gestational-age z score chart for the assessment of maternal weight gain in pregnancy. Am J Clin Nutr. 2013;97(5):1062–7. doi: 10.3945/ajcn.112.051706 23466397 PMC3625243

[36] Carrilho TRB, MR K, Rodrigues Farias D, Freitas Costa NC, Araújo Batalha M, ER M, et al. Agreement between self-reported pre-pregnancy weight and measured first-trimester weight in Brazilian women. BMC Pregnancy Childbirth. 2020;20(1):734. doi: 10.1186/s12884-020-03354-4 33243188 PMC7690094

