# Supplementary figures and images for: Establishment of a Latin American dataset to enable the construction of gestational weight gain charts for adolescents
Source: PLoS One. 2024 Jan 26;19(1):e0296981. doi: 10.1371/journal.pone.0296981 (PMC10817143; doi:10.1371/journal.pone.0296981)

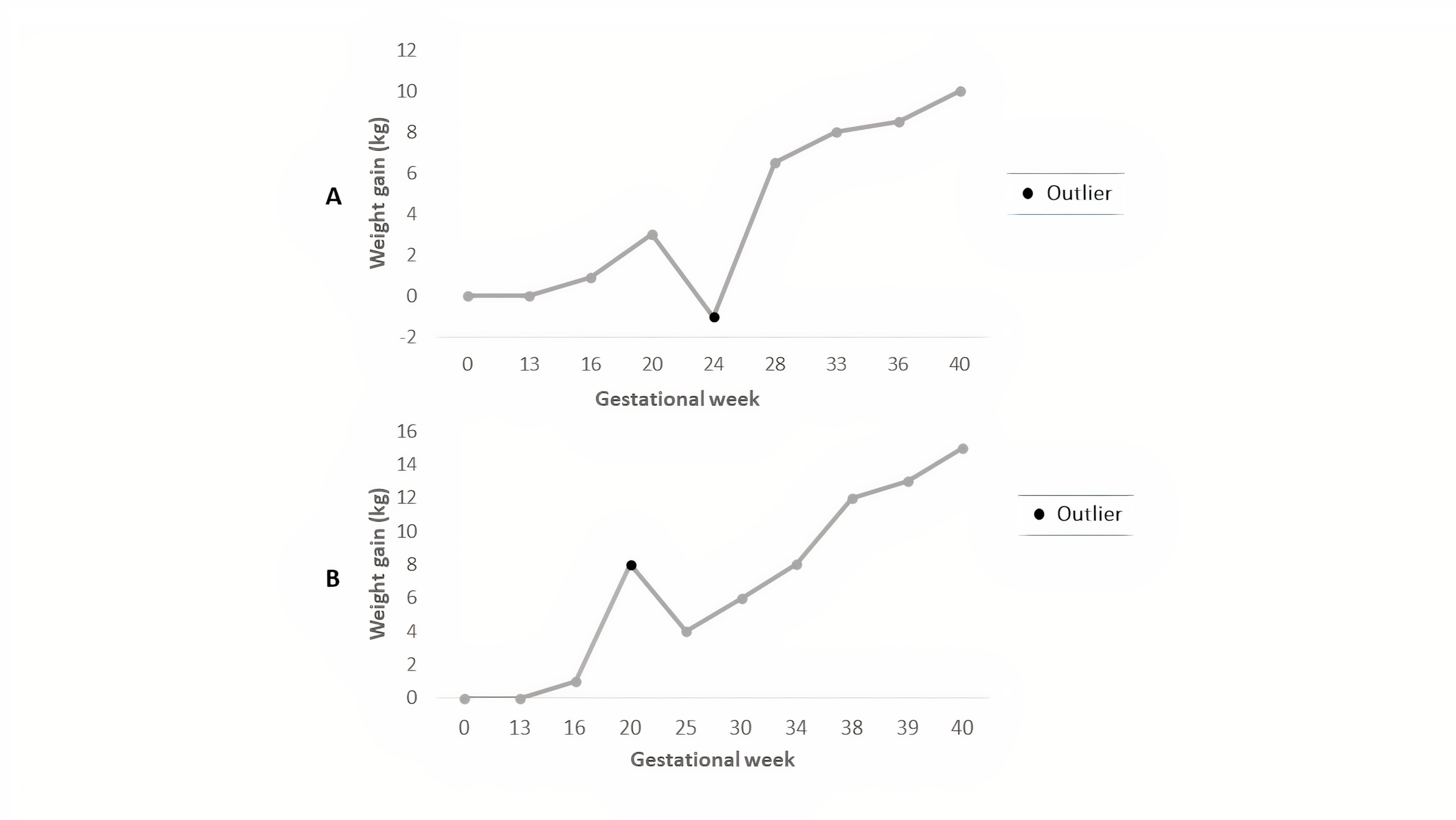

Supplement: S1 Fig — (TIF) [file pone.0296981.s001.tif]

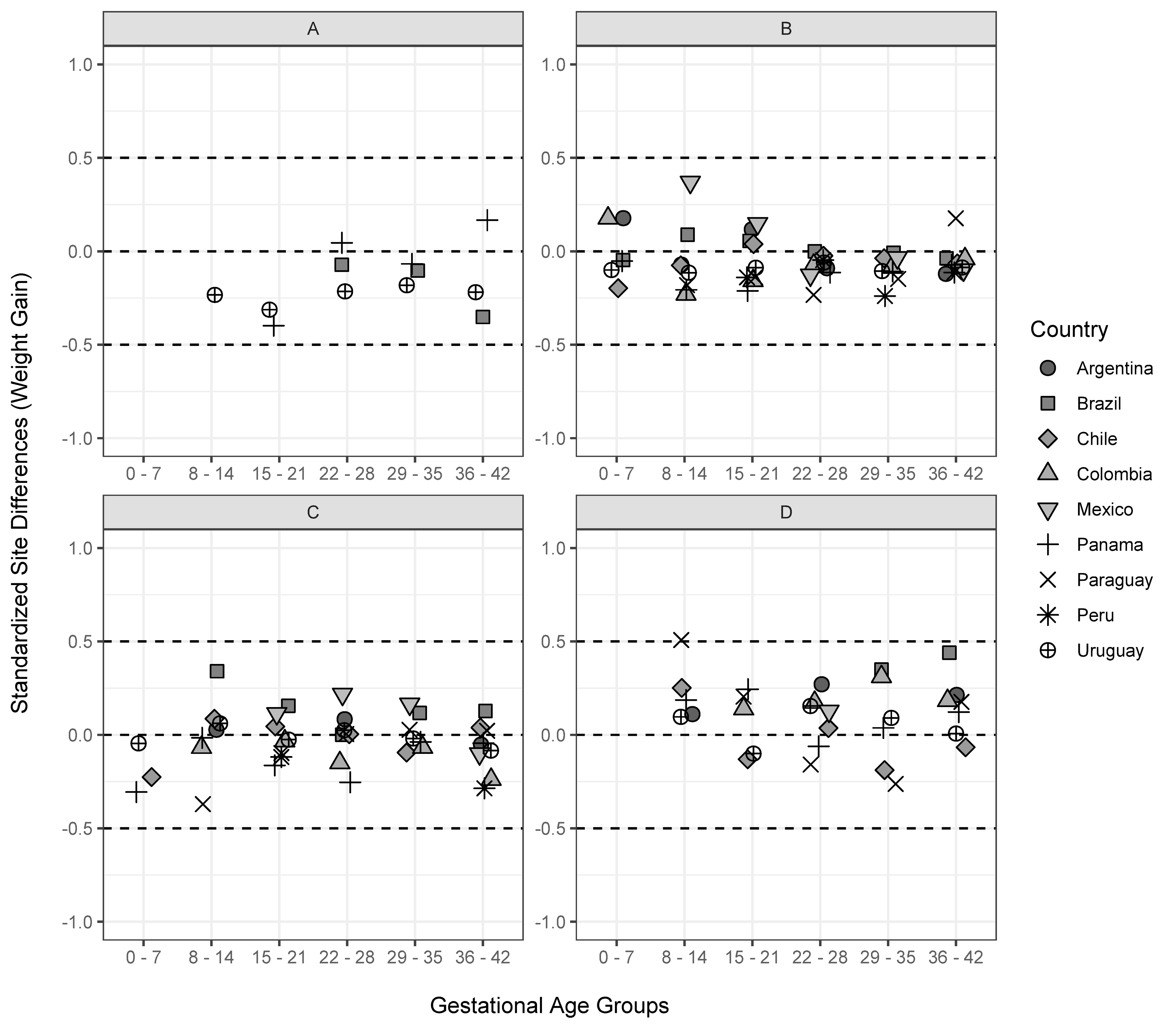

Supplement: S2 Fig — Standardized Site Differences (SSD) for gestational weight gain (kg) according to country and gestational age group (weeks) removing datasets with n ≤ 10 in the selected time intervals: A. underweight (BMI/age <-2 SD); B. normal weight (BMI/age ≥ -2 SD and ≤ +1 SD); C. overweight (BMI/age > +1 SD and ≤ +2SD); D. obesity (BMI/age > +2 SD). (TIF) [file pone.0296981.s002.tif]
