# Supplementary material for: Establishment of a Latin American dataset to enable the construction of gestational weight gain charts for adolescents
Source: PLoS One. 2024 Jan 26;19(1):e0296981. doi: 10.1371/journal.pone.0296981 (PMC10817143; doi:10.1371/journal.pone.0296981)
Supplement: S1 File — (DOCX) [file pone.0296981.s003.docx]

**Supplementary tables**

**S1 Table**. **Origin and source of the databases by country.**

| Country | Dates | Origin | n | % |
| --- | --- | --- | --- | --- |
| Argentina | 2012-2019 | Medical records | 545 | 8,5 |
| Brazil | 2003-2010 | Research project and medical records | 254 | 4,0 |
| Chile | 2013-2015 | Research project | 324 | 5,1 |
| Colombia | 2009-2021 | Research project and Information system | 295 | 4,6 |
| Mexico | 2012-2020 | Medical records | 119 | 1,9 |
| Panamá | 2011-2020 | Information system | 2042 | 31,8 |
| Paraguay | 2015-2020 | Medical records | 396 | 6,2 |
| Perú | 2016-2018 | Medical records | 91 | 1,4 |
| Uruguay | 2008-2010 | Research project and information system | 2348 | 36,6 |

**S2 Table.** **Distribution of gestational weight gain according to pre-pregnancy body mass index (BMI), country and trimester of pregnancy before the imputation process.**

| Country | Underweight  (<-2 SD) | | Normal  (≥ -2 SD and ≤ +1 SD) | | Overweight  (> +1 SD to ≤ +2SD) | | Obesity  (> +2 SD) | |
| --- | --- | --- | --- | --- | --- | --- | --- | --- |
|  | n | Median (IQR) | n | Median (IQR) | n | Median (IQR) | n | Median (IQR) |
| 1^st^ pregnancy trimester | | | | | | | | |
| Argentina | 4 | -2.0 (-2.0 - -0.2) | 366 | 1.3 (0 – 3.0) | 111 | 0.5 (-1.6 – 2.0) | 31 | 0.1 (-3.6 – 2.0) |
| Brazil | 7 | 7.5 (3.7 – 8.9) | 162 | 1.6 (0 – 2.9) | 30 | 1.1 (-1.6 – 2.4) | 5 | 1.6 (0.5 – 3.0) |
| Chile | - | - | 183 | 1.0 (-0.3 – 2.5) | 65 | 0.9 (-0.1 – 2.8) | 22 | 2.2 (0 – 2.6) |
| Colombia | 8 | 2.2 (1.0 – 2.8) | 173 | 0 (-0.4 – 1.0) | 44 | 0 (-0.5 – 0.4) | 6 | 0 (-2.2 – 1.1) |
| Mexico | - | - | 9 | 2.7 (1.2 – 3.5) | 3 | 1.0 (-2.0 – 1.4) | - | - |
| Panama | 5 | 1.2 (0.9 – 7.0) | 139 | 0 (-1.0 – 1.0) | 58 | 0.4 (0 – 1.9) | 16 | 0.4 (-0.8 – 1.5) |
| Paraguay | - | - | 97 | 1.0 (0 – 3.0) | 34 | 0.1 (0 – 2.0) | 14 | 0 (-3.2 – 0) |
| Peru | - | - | - | - | - | - | - | - |
| Uruguay | 15 | 2.5 (0.8 – 7.6) | 1132 | 1.1 (0 – 3.0) | 145 | 0.6 (-0.8 – 2.0) | 33 | 1.3 (0.3 – 2.5) |
|  |  |  |  |  |  |  |  |  |
| 2^nd^ pregnancy trimester | | | | | | | | |
| Argentina | 4 | 6.8 (5.2 – 8.4) | 397 | 6.0 (4.0 – 8.2) | 128 | 4.4 (1.0 – 6.6) | 41 | 5.0 (0 – 7.0) |
| Brazil | 21 | 9.9 (8.0 – 13.4) | 645 | 5.1 (2.5 – 7.6) | 85 | 3.7 (1.3 – 6.6) | 13 | 2.7 (-1.6 – 5.8) |
| Chile | 3 | 2.8 (2.8 – 5.0) | 509 | 4.7 (1.8 – 7.6) | 213 | 3.2 (0.1 – 6.8) | 55 | 3.7 (1.6 – 6.5) |
| Colombia | 16 | 6.2 (3.9 – 9.0) | 600 | 3.5 (1.0 – 6.0) | 146 | 1.2 (0 – 4.0) | 33 | 1.8 (0 – 3.5) |
| Mexico | 1 | 6 (-) | 231 | 4.7 (2.2 – 7.0) | 56 | 4.5 (-0.5 – 6.2) | 17 | 2.5 (0 – 5.0) |
| Panama | 32 | 3.6 (1.4 – 8.1) | 1452 | 0.7 (0 – 3.1) | 732 | 0.2 (0 – 1.0) | 192 | 0 (0 – 0.8) |
| Paraguay | 11 | 7.0 (5.0 – 11.0) | 535 | 3.5 (1.0 – 6.6) | 121 | 1.5 (0 – 4.0) | 42 | 2.0 (0.1 – 4.8) |
| Peru | - | - | 70 | 4.0 (1.0 – 6.0) | 18 | 0 (-1.0 – 2.8) | 3 | 0 (0 – 0.5) |
| Uruguay | 97 | 6.9 (4.0 – 11.0) | 4309 | 4.7 (2.0 – 7.5) | 678 | 3.0 (0.7 – 6.5) | 136 | 3.0 (0 – 6.0) |
|  |  |  |  |  |  |  |  |  |
| 3^rd^ pregnancy trimester | | | | | | | | |
| Argentina | 4 | 12.2 (8.0 – 16.6) | 385 | 13.1 (11.0 – 16.4) | 120 | 11.4 (9.0 – 15.3) | 37 | 13.0 (8.0 – 15.5) |
| Brazil | 28 | 17.2 (14.8 – 19.2) | 863 | 12.0 (9.0 – 15.0) | 133 | 11.8 (6.9 – 15.2) | 30 | 10.6 (4.3 – 12.2) |
| Chile | 1 | 9.0 (-) | 634 | 12.0 (9.1 – 15.5) | 255 | 10.6 (6.8 – 14.5) | 63 | 9.0 (5.5 – 14.2) |
| Colombia | 7 | 12.0 (9.5 – 13.5) | 606 | 10.0 (7.1 – 13.0) | 127 | 7.4 (5.0 – 9.8) | 40 | 9.6 (5.9 – 11.1) |
| Mexico | 6 | 10.8 (10.5 – 11.8) | 390 | 11.0 (7.5 – 14.0) | 75 | 8.6 (4.0 – 11.2) | 17 | 7.0 (1.0 – 9.0) |
| Panama | 28 | 11.0 (8.8 – 14.1) | 1482 | 8.4 (5.5 – 11.9) | 730 | 7.0 (4.0 – 10.2) | 190 | 7.0 (3.6 – 9.5) |
| Paraguay | 11 | 17.0 (13.5 – 18.8) | 504 | 9.6 (6.0 – 14.0) | 133 | 7.0 (3.0 – 11.0) | 39 | 6.0 (3.0 – 11.0) |
| Peru | - | - | 70 | 12.0 (10.0 – 15.0) | 18 | 8.0 (4.0 – 15.8) | 3 | 11.0 (10.0 – 13.0) |
| Uruguay | 132 | 12.1 (9.6 – 15.6) | 5727 | 11.5 (8.2 – 15.0) | 896 | 10.0 (6.2 – 14.5) | 186 | 9.6 (6.0 – 13.0) |

**S3 Table. Distribution of gestational weight gain according to pre-pregnancy body mass index (BMI) and country in the first pregnancy trimester after the imputation process.**

| Country | Underweight  (<-2 SD) | | Normal  (≥ -2 SD and ≤ +1 SD) | | Overweight  (> +1 SD to ≤ +2SD) | | Obesity  (> +2 SD) | |
| --- | --- | --- | --- | --- | --- | --- | --- | --- |
|  | n | Median (IQR) | n | Median (IQR) | n | Median (IQR) | n | Median (IQR) |
| Argentina | 4 | -2 (-2 - -0.2) | 373 | 1.3 (0 - 3) | 124 | 0.7 (-1.5 - 2.1) | 34 | 0.0 (-3.2 – 2.0) |
| Brasil | 11 | 5 (2.9 - 8.1) | 209 | 1.7 (0 - 3.2) | 46 | 1.4 (-0.3 - 2.4) | 7 | 1.6 (-0.3 - 3.1) |
| Chile | 1 | -0.07 (-) | 215 | 1 (-0.1 - 2.5) | 111 | 0.9 (-0.4 - 3) | 26 | 2.2 (0 - 2.7) |
| Colombia | 9 | 2.5 (2.0 - 3.6) | 207 | 0 (-0.5 - 1) | 77 | 0 (-0.5 - 1) | 7 | 0 (-1.5 - 1) |
| México | 1 | 1.37 (-) | 11 | 2.3 (0.6 - 3.5) | 23 | 0.5 (-0.9 - 1.5) | 0 | - |
| Panama | 28 | 2.0 (1.2 - 3.5) | 268 | 0 (-0.3 - 1) | 713 | 0.7 (-0.5 - 2.0) | 27 | 0.4 (-0.9 - 1.5) |
| Paraguay | 5 | 2.7 (2.0 - 3.3) | 129 | 1 (0 - 3) | 88 | 0.4 (-0.6 – 2.0) | 14 | 0 (-3.2 - 0) |
| Peru | 0 | - | 5 | 0 (0 - 0.8) | 18 | 0.2 (-0.5 - 2.4) | 1 | 1 (-) |
| Uruguay | 55 | 3.1 (1.9 - 5.8) | 1375 | 1.2 (0 - 3) | 377 | 0.8 (-0.8 - 2.4) | 42 | 1.4 (0 - 2.4) |
